# Case Report: Vasospastic angina presenting as phantom odor perception

**DOI:** 10.3389/fcvm.2024.1416149

**Published:** 2024-07-04

**Authors:** Haroon Zubair, Valentin Suma, Faisal Masood, M. Fuad Jan, Tanvir Bajwa, Babak Haddadian

**Affiliations:** Aurora Cardiovascular and Thoracic Services, Aurora Sinai/Aurora St. Luke’s Medical Centers, Aurora Health Care, Milwaukee, WI, United States

**Keywords:** odor, perception, vasospastic angina, case report

## Abstract

**Background:**

Vasospastic angina usually presents with intermittent episodes of chest pain. It can rarely be associated with the perception of phantom odors.

**Case summary:**

A 69-year-old woman presented for evaluation of intermittent shortness of breath and chest pain. She reported that she often experienced an abnormal smell sensation just prior to the event. The patient had abnormal smell sensation and shortness of breath at the initiation of exercise stress echocardiography with transient electrocardiographic changes and new regional wall motion abnormalities. Subsequent invasive coronary angiography showed no obstructive epicardial coronary artery disease. The patient was started on calcium channel blocker therapy with resolution of symptoms.

**Conclusion:**

Phantom odor perception has been rarely reported as an angina-equivalent symptom. Clinicians should have a high index of suspicion in patients presenting with atypical anginal symptoms.

## Introduction

Vasospastic angina is a variant form of angina that usually occurs at rest (1). It is associated with transient electrocardiographic (ECG) changes which may resolve spontaneously or with the administration of nitroglycerin. Affected patients tend to be younger and have less risk factors for coronary artery disease as opposed to those with angina secondary to atherosclerosis. The exact prevalence of this disease is unknown and it is likely underdiagnosed with subsequent undertreatment (2). Prompt and correct identification is of great importance as its presence has been shown to identify patients at increased risk for myocardial infarction and death (3); furthermore, this may help prevent repeated and frequently invasive diagnostic evaluations, help reduce health care cost and most importantly, promote patient-tailored management. A rare association between angina and phantom odor perception has been previously described in literature (4). We describe the case of a 69-year-old woman who was evaluated for similar phantom odor perception and diagnosed with vasospastic angina as the likely etiology of her symptoms.

## Case summary

A 69-year-old African American female with a medical history significant for hypertension, migraine headaches, and tobacco use, was seen in an urgent care facility for intermittent episodes of shortness of breath and chest discomfort. At home she was on lisinopril, and she denied any history of alcohol or drug abuse, including cocaine and marijuana. She had no history of psychiatric illness. She was referred to the emergency room for further evaluation. The patient described these episodes as occurring at rest and with exertion, lasting about 10–15 min, and self-resolving. She noted that she could anticipate the onset of shortness of breath and chest pain as this was preceded by a phantom smell perception. She described it as noticing a smell, which people around her did not notice. Upon further questioning, the patient stated that she had been having similar episodes of dyspnea for about nine years. These episodes always started with an odor hallucination as described above. This could vary between unpleasant smells like burnt objects or pleasant ones such as perfume or sweets. Invariably, the phantom odor would be followed by shortness of breath and diaphoresis. Initially, these episodes were very infrequent and occurring a few times a year. However, for the past few months, the patient stated that she had been experiencing progressively worsening symptoms, starting with phantom odor perception with subsequent shortness of breath and chest discomfort. The presenting episode lasted longer than previous episodes. Hence, she presented to urgent care for further evaluation. Of note, further questioning revealed that when she was in her 20s, she used to have severe migraine attacks with an aura described as flashing lights. Her daughter also had a medical history significant for migraine headaches.

Physical examination revealed elevated blood pressure of 150/72 mmHg and otherwise a normal cardiac examination with a regular rate and rhythm and normal heart sounds. Initial ECG showed normal sinus rhythm with no acute ischemic abnormalities. Laboratory data were unremarkable, including a comprehensive metabolic profile, complete blood count, pro-B-type natriuretic peptide, and high-sensitivity troponin. Chest x-ray did not reveal any acute cardiopulmonary process. Serial high-sensitivity troponin values were checked and remained within normal limits.

**Figure 1:**
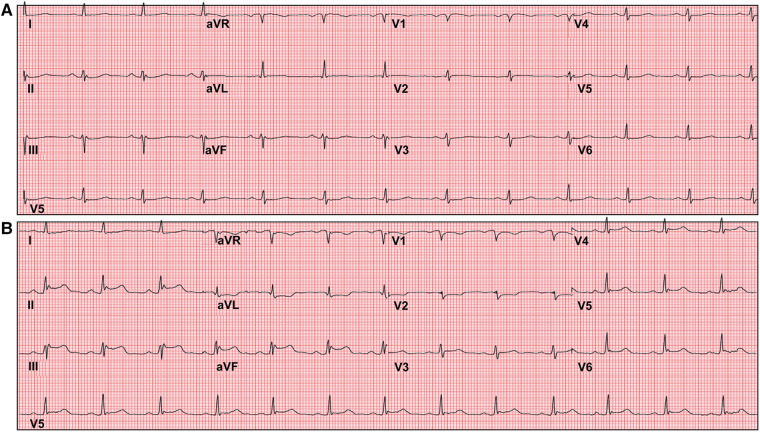
Exercise tolerance test electrocardiogram (ECG). Panel (**A**) shows baseline ECG before initiation of the stress test. Panel (**B**) shows significant ST segment elevations in inferior leads with reciprocal ST depressions in leads I, aVL and V1-2 shortly after inception of exercise.

**Figure 2:**
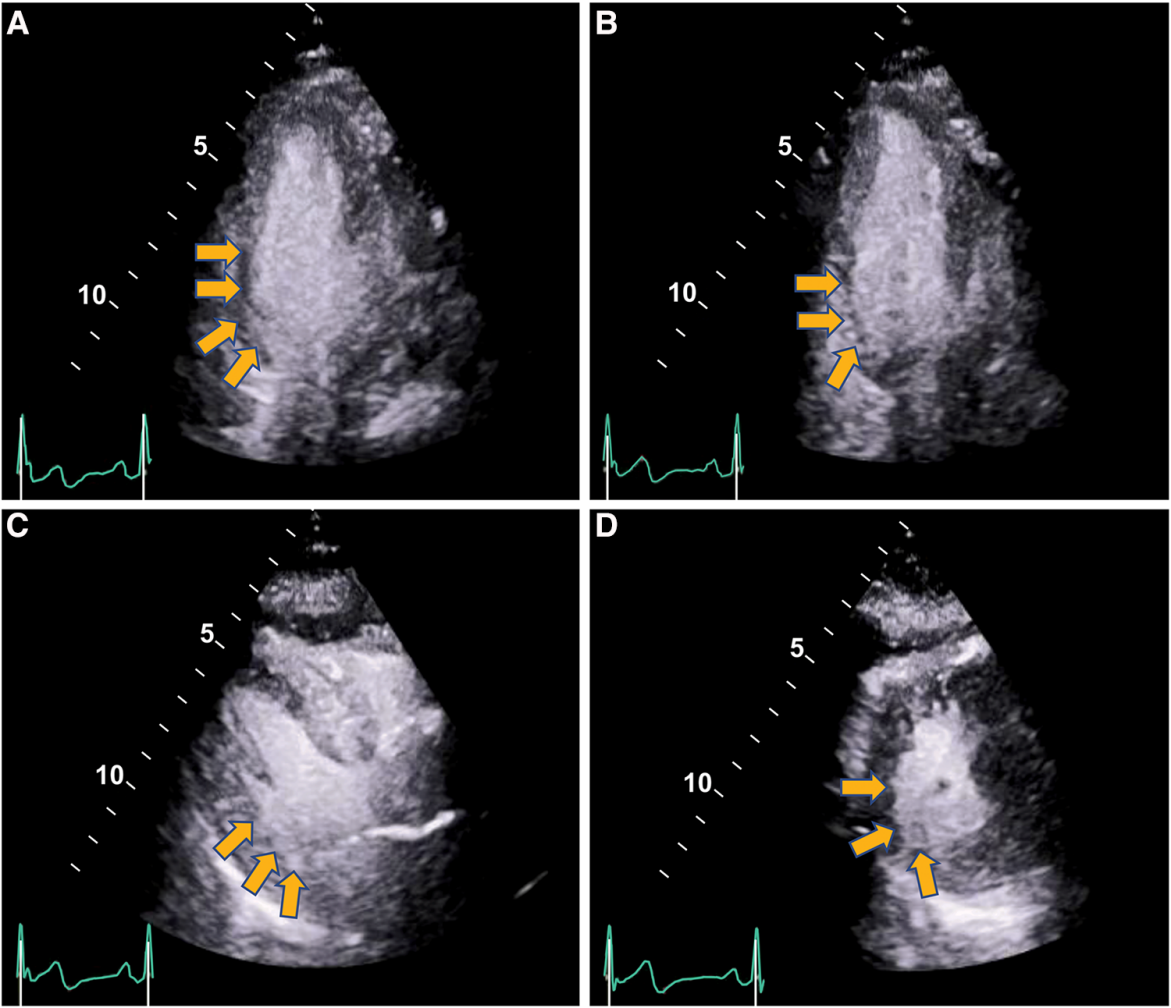
Regional wall motion abnormalities with severe hypokinesis of the basal infero-septal, basal, and mid inferior and infero-lateral segments seen on stress echocardiography during anginal episode (yellow arrows) (end-systolic frames shown). Panel (**A**) Apical 4-chamber. Panel (**B**) Apical 2-chamber. Panel (**C**) Parasternal long axis. Panel (**D**) Parasternal short axis. (Supplementary Videos S1, S2).

The care team made the clinical decision to proceed with cardiac stress testing for further risk stratification. Within 15–30 s of the initiation of exercise, the patient started to notice phantom odor perception which was not observed by the staff standing around the patient; this was immediately followed by ECG changes of significant ST elevations in the inferior leads with reciprocal changes in leads I, aVL and V1-2 (Figure 1), associated with chest discomfort and shortness of breath. The test was instantly aborted, and the patient was assisted back to a supine position. Echocardiography images were obtained immediately showing new left ventricular regional wall motion abnormalities that were concordant with the electrocardiographic changes (Figure 2, Supplementary Video S1). The ST elevations resolved within 40 s, and repeat echocardiographic images also showed resolution of the regional wall motion abnormalities. This was shortly followed by another similar self-limited episode of transient ST elevations on ECG associated with anginal symptoms. Owing to ECG changes concerning for acute coronary plaque rupture, the patient was transported emergently to the cardiac catheterization suite. Invasive coronary angiography showed sluggish flow in the right coronary artery but no obstructive epicardial coronary artery disease (Supplementary Video S2).

The transient ECG and echocardiographic changes were witnessed during the anginal episodes. Hence, the patient was clinically diagnosed with vasospastic angina, most likely epicardial in the setting of significant inferior ST segment elevations on ECG. Further provocative testing was deemed not indicated at the time. The patient was started on treatment with verapamil, a calcium channel blocker medication. Smoking cessation was discussed in detail. At the one-month outpatient follow-up, the patient denied any further cardiac symptoms.

## Discussion

Vasospastic angina, also known as Prinzmetal angina, was described in 1959 (5). However, its true prevalence remains largely undetermined. A recent systematic meta-analysis of 56 studies comprising 14,427 patients with suspected coronary artery disease who underwent invasive coronary angiography and were found to have nonobstructive epicardial coronary artery disease, reported that half of these patients were found to have coronary microvascular dysfunction and/or coronary spasm (pooled prevalence of epicardial vs. microvascular spasm of approximately 40% vs. 24%, respectively (6). Only chronic microvascular disease was reported to be more prevalent in females. It has been hypothesized that vasospastic angina may be the coronary manifestation of a generalized vasomotor disorder. As such, it may be associated with migraine headache and Raynaud phenomenon (7). Our patient also described a past medical history significant for migraine headaches with aura. Though it was interesting to note that she stopped having these migraine attacks decades prior to the initiation of angina symptoms.

Phantosmia refers to an odor perception which occurs in the absence of an odor stimulus (8). Such odor perceptions are usually unpleasant, including the smell of burnt toast, tobacco smoke, chemical or stale food. Rarely, they are also described as pleasant smells, such as sweets. Phantom odor perception is hypothesized to arise from aberrant peripheral olfactory sensory neurons or anomalies with signaling perception centers in the brain (4). Common etiologies of phantosmia includes post-traumatic and post-infectious olfactory dysfunction. It may also occur in neuropsychiatric, sinonasal, or idiopathic olfactory dysfunction (9). The association between phantom odor perception and vascular conditions among older adults has been described previously in a cross-sectional analysis of 7,417 US adult patients aged 40 years and older (4). In a multivariable-adjusted analysis, patients aged 60 years or above with symptoms of angina were about three times more likely to experience phantom odor perception. Adults with higher blood pressure were also more likely to report phantom odors. Concordantly, the patient that we described was 69 years old and had a medical history significant for hypertension. While the etiology of phantom odor perception in this scenario is not known, it has been hypothesized that perhaps coexisting vascular conditions in the olfactory cortex could be explanatory. It is possible that abnormal blood flow within the olfactory cortex in the setting of transient coronary spasm could be contributory in patients such as ours.

Patients with vasospastic angina often present with chronic recurring episodes of angina, which are usually responsive to nitroglycerin. The etiology is thought to be due to coronary endothelial dysfunction. Vascular hyperreactivity and resulting vasospasm may occur, leading to symptoms and ischemic ECG changes. Provocative testing with acetylcholine may be pursued for further evaluation of coronary vasospasm, though it is rarely performed. Such testing has been shown to be a safe way to assess for coronary vasomotor function (10). However, this was not clinically necessary for our patient owing to documented transient ECG and echocardiographic changes during anginal episodes.

The treatment for vasospastic angina usually comprises of therapy with a calcium channel blocker alone or in combination with nitrates. Treatment of angina in the setting of nonobstructive coronary artery disease due to suspected vasomotor dysfunction with diltiazem has shown a reduction in the prevalence of epicardial spasms (11). Tobacco cessation and lifestyle modification should also be advised.

## Data Availability

The original contributions presented in the study are included in the article/**Supplementary Material**, further inquiries can be directed to the corresponding author.
